# BCG enables protection against malaria through adjuvanting an adenovirus-vectored vaccine

**DOI:** 10.1038/s41541-026-01506-9

**Published:** 2026-06-24

**Authors:** Sören Reinke, Marta Ulaszewska, Qi Su, Lee Sims, Romain Guyon, Adam Truby, Gundra Raissar, Jelena S. Bezbradica, Joannah R. Fergusson, Anita Milicic

**Affiliations:** 1 The Jenner Institute, Nuffield Department of Medicine, University of Oxford, Oxford, UK; 2 Kennedy Institute of Rheumatology Research, Nuffield Department of Orthopaedics, Rheumatology and Musculoskeletal Sciences, Medical Sciences Division, University of Oxford, Oxford, UK; 3 Botnar Research Centre, Institute of Biomedical Engineering, University of Oxford, Oxford, UK; 4 Present Address: Pandemic Sciences Institute, Nuffield Department of Medicine, University of Oxford, Oxford, UK

**Keywords:** Diseases, Immunology, Microbiology

## Abstract

BCG vaccination against TB may also reduce malaria incidence through non-specific innate immune activation. We investigated whether giving BCG alongside an adenoviral-vectored malaria vaccine improves protection against malaria in mice. Co-immunisation broadened and strengthened the early innate cytokine responses, enabling strong CD8^+^ T cell-dependent protection. Our findings suggest co-immunisation with BCG and adenoviral vector as a promising strategy to combat both TB and malaria in double-endemic regions.

The tuberculosis (TB) vaccine Bacillus Calmette-Guérin (BCG) has well-documented off-target benefits against a broad range of infections, including malaria, sepsis, leprosy, SARS-CoV-2, and even as therapy in bladder cancer^1^. These non-specific effects are believed to be underpinned by epigenetic imprinting of myeloid cells^2^, resulting in enhanced secretion of pro-inflammatory cytokines such as TNF and IL-1β for up to a few months post BCG vaccination^3^.

BCG-induced non-specific immunity against malaria^4–8^ is of particular significance as the endemic geographic regions of TB and malaria largely overlap^9^. Field studies in sub-Saharan Africa have shown associations between BCG vaccination and reduced malaria prevalence in children under five, and seasonal reductions in all-cause neonatal mortality^9,10^. However, a study of controlled human malaria infection following BCG vaccination failed to demonstrate non-specific BCG-mediated protection, highlighting the ongoing uncertainty regarding the role of BCG vaccination in immunity against malaria^11^. To date, the protective effect of BCG has not been investigated in the context of malaria vaccination. In view of its specific adjuvant-like properties through pathogen recognition receptor (PRR) activation, along with the off-target effects on malaria, it is important to consider vaccination strategies that administer malaria and BCG vaccines at the same time or in temporal proximity, to exploit the full potential of BCG in reducing both malaria and TB burden in double-endemic regions.

This study explores the effect of BCG on the immune response elicited by an adenoviral-vectored malaria vaccine (human Adenovirus serotype 5, hereafter referred to as Ad), in a mouse model of malaria. Notably, the adenoviral vector in this study was used as proof of concept to assess whether and how the efficacy of vectored vaccines can be enhanced with a concurrent administration of the BCG vaccine. The Ad construct encodes an immunodominant CD8^+^ T cell epitope Pb9 from *Plasmodium berghei* (*P. berghei*) circumsporozoite protein, which mediates protection against liver-stage malaria in this mouse model^12–14^. The vaccines were administered into contralateral limbs to prevent any unknown vaccine–vaccine interactions and exacerbated reactogenicity at the injection site, and to preserve the translational aspect of the study. Contralateral BCG vaccination has previously been safely deployed in the clinic^15^.

## Ad+BCG co-administration enables protection against malaria in mice

**Fig. 1:**
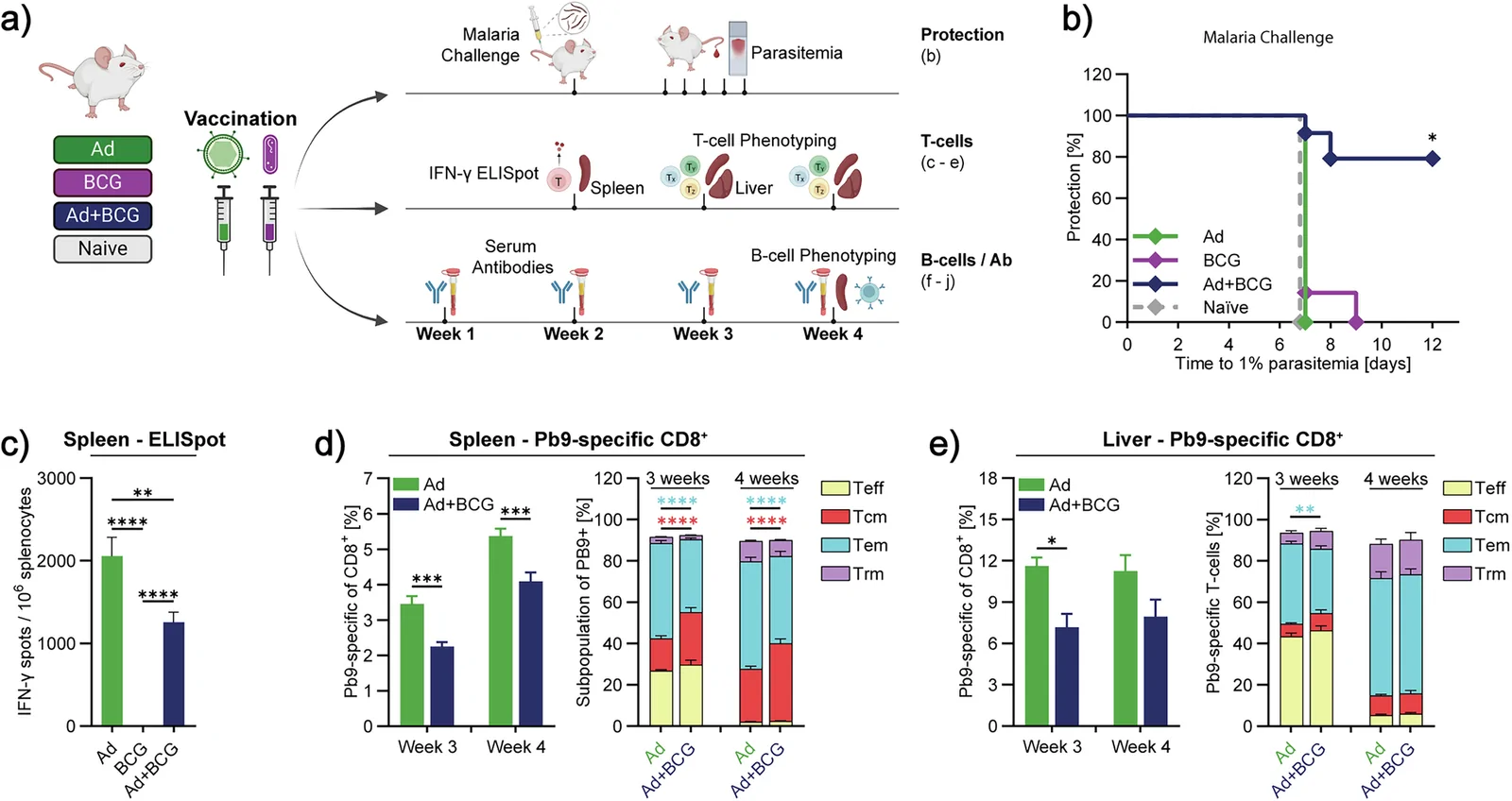
Ad + BCG co-administration enables protection against malaria in mice. **a** Summary of experimental design, indicating corresponding figure panels. Vaccine dose per mouse: 0.5 × 10^5^ CFU for BCG (i.d. or i.m.) and 5 × 10^7^ i.u. for AdHu5-TIPeGFP (i.m.). **b** Survival post-malaria challenge: BALB/c mice were vaccinated and challenged as in (**a**). Parasitaemia was assessed by daily blood smears, with 1% parasitaemia taken as the end-point^33^. Ad + BCG vs. Ad or BCG alone: *p* < 0.01, pairwise Fisher test with Bonferroni correction (*n* ≥ 7). **c** IFN-γ producing CD8^+^ T cells in the spleen 2 weeks post-vaccination, restimulated with the Pb9 peptide for 20 h ex vivo. Data show the mean + SEM (*n* = 8); one-way ANOVA with Bonferroni’s correction for multiple comparisons; **p < 0.01; ****p < 0.0001. **d**, **e** T cell phenotyping in spleen and liver after vaccination and harvest as in (**a**). Left-hand panels show the percentage of antigen (Ag) specific CD8^+^ T cells 3 or 4 weeks after vaccination. Right-hand panels show the distribution of T cell subpopulations within the Pb9^+^, CD8^+^ compartment. Gating strategy as in Supplementary Fig. 1d. Mean + SEM (*n* = 6); two-way ANOVA with Bonferroni’s correction for multiple comparisons; *p < 0.05; **p < 0.01; ***p < 0.001; ****p < 0.0001.

Mice were immunised with a low sub-protective dose of Ad, BCG alone, or Ad+BCG (administered at different sites), and challenged with *P. berghei* malaria parasites 2–4 weeks later (Fig. 1a). The low dose of Ad allows us to observe any BCG-mediated enhancement of protection, given that SPF-housed mice are known to have an augmented response to vaccination and are more readily protected than humans^16^. Mice vaccinated with Ad or BCG alone had no protection against malaria (Fig. 1b), whereas 80% of the mice that received both vaccines developed strong sterile immunity (Fig. 1b). This suggests that BCG is adjuvanting the adaptive, Pb9 CD8^+^ T cell-driven response against malaria either through innate immune imprinting or by directly adjuvanting the innate response to the Ad-vectored vaccine. To assess the role of myeloid cell imprinting^17^ in protection, mice were vaccinated with BCG two or four weeks prior to Ad vaccination. These regimens resulted in a lower survival rate compared to Ad-BCG co-administration (Supplementary Fig. 1a), suggesting that the key element in protection against malaria in this model is the direct BCG-adjuvanting effect of the Ad vaccine.

## Ad+BCG co-administration modulates the T and B cell compartments

To mechanistically dissect the protective immune response and the additive benefit of BCG and Ad in the context of liver-stage malaria, we phenotyped splenic and hepatic CD8^+^ T cells^18^ and analysed antibody (Ab) responses after vaccination with Ad, BCG and Ad+BCG (Fig. 1a). Of note, a reduced percentage of Pb9^+^ specific CD8^+^ T cells was observed in the spleens and livers of mice receiving Ad+BCG compared to Ad alone (Fig. 1c–e and Supplementary Fig. 1b, c), replicating our previous findings with adjuvanted Ad vaccine in malaria protection^13^. Vaccination with BCG also affected the distribution of Pb9^+^ CD8^+^ T cell memory subpopulations, resulting in a higher proportion of central memory T cells (Tcm) and reduction in effector memory T cells (Tem) than with the Ad vaccine alone (Fig. 1d). Importantly, immunisation with BCG did not affect the CD8^+^ T cell surface density of the liver-homing cell marker CXCR6^19^ (Supplementary Fig. 1b, c), which has previously been associated with protection against liver-stage malaria in mice, and which we found to be more abundant on Pb9-specific T cells compared to the Pb9^−^CD8^+^ T cell subset (Supplementary Fig. 1b, c).

Given that the adaptive immunity against malaria generated by this vaccine is reliant solely on the Pb9 T cell epitope, the observed reduction in total antigen (Ag)-specific CD8^+^ T cells suggests that BCG-induced perturbation of T cell memory subsets may be of significance, with the protection driven by the type (rather than the total number) of Ag-specific CD8^+^ T cells. Antigen-specific Tcm, which were enriched after Ad + BCG vaccination, have also previously been demonstrated to enhance sterile protection in this model^13^. Indeed, the efficiency of the CD8^+^ T cell response relies on the quantity/density of the presented Ag, as well as the quality of co-stimulation by the myeloid antigen- presenting cells, which are targets of BCG activation^20^. These findings are indicative of a qualitative T cell improvement which is associated with enhanced protection.

To broaden the relevance of this immunisation approach to other pathogen indications, we also investigated the effect of Ad+BCG co-administration on B cells and antibody responses, and observed a reduction in the total number of germinal centre B cells and plasma cells compared to Ad alone (Supplementary Fig. 2a–c). Likewise, immunisation with Ad+BCG led to a slight reduction in the overall numbers of plasmablasts and memory B cells, compared to Ad alone (Supplementary Fig. 2d–f). To investigate if the addition of BCG might have resulted in a weakened Ab response against the Ad vaccine, which encodes GFP as a surrogate Ag, we measured anti-GFP IgG titres post-vaccination. Akin to our T cell data, additional immunisation with BCG did not affect Ag-specific IgG titres despite a reduction in the number of circulating lymphocytes (Supplementary Fig. 2h). In parallel, the addition of the Ad vaccine enhanced the anti-BCG humoral response, and increased seroconversion against BCG in the Ad+BCG group compared to BCG alone. (Supplementary Fig. 2i). These findings suggest a reciprocal adjuvanting effect between Ad and BCG, indicating that simultaneous vaccination with BCG and viral-vectored vaccines could enhance protection against both TB and the disease targeted by the vectored vaccine. Furthermore, proportional analyses of ELISA readouts revealed that BCG administration modestly skewed the responses against the vector-expressed antigens towards T_H_1-type immunity, which is in line with recent findings^21^. This induced bias may be beneficial for vaccine strategies that aim to preferentially elicit strong T_H_1-type immune responses.

## BCG broadens the systemic innate cytokine milieu

**Fig. 2:**
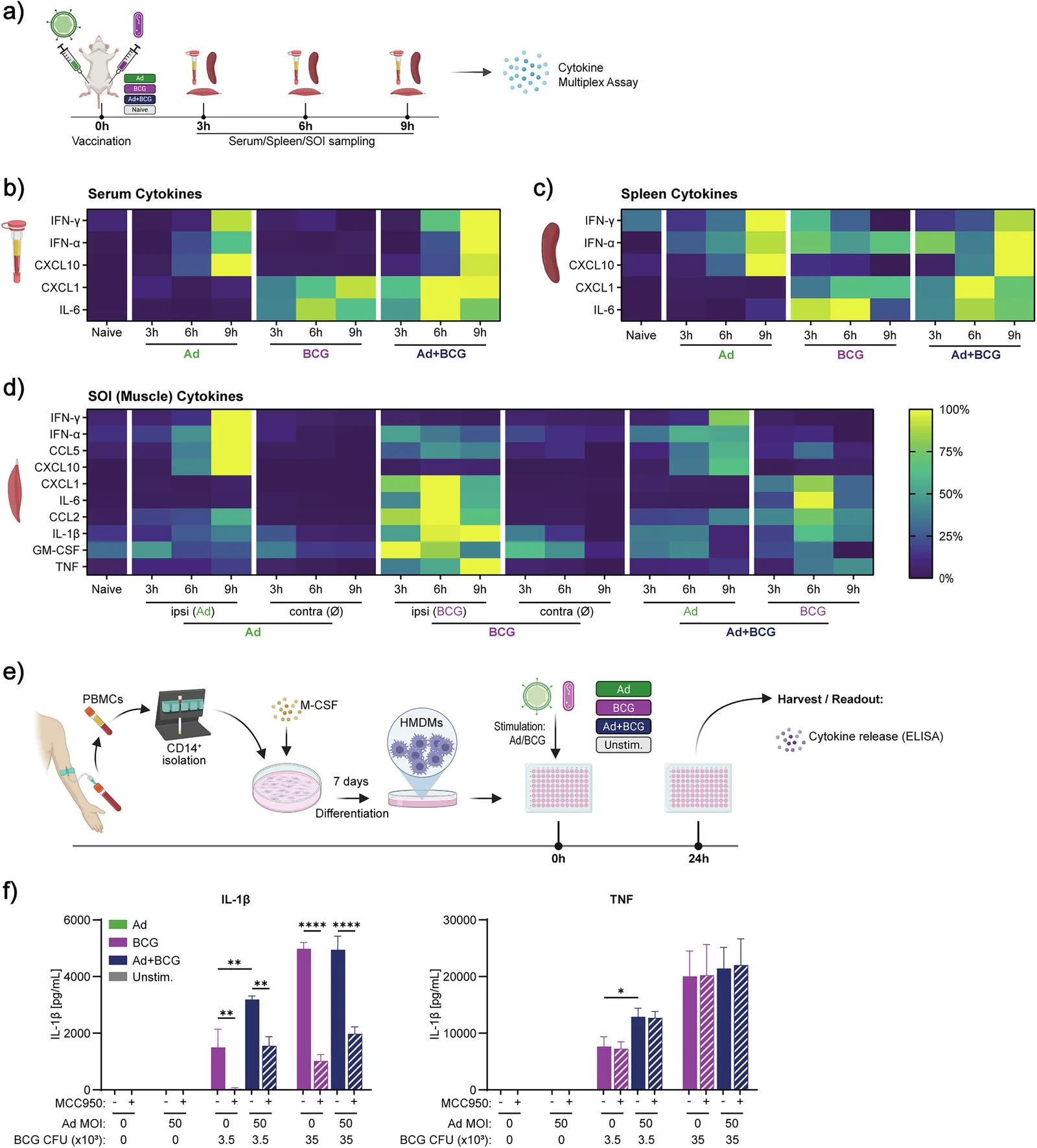
BCG broadens the systemic innate cytokine milieu. **a** Summary of the experimental design for the data in (**b**–**d**). Vaccine dose per mouse: 0.5 × 10^5^ CFU for BCG and 5 × 10^7^ i.u. for AdHu5-TIPeGFP. In the Ad + BCG group, Ad was injected into the right leg and BCG into the left leg. **b**–**d** Indicated cytokines were measured using LEGENDplex^TM^ MU Anti-Virus Response Panel. Heatmaps show relative cytokine levels detected in **b** the serum, **c** the spleen, and **d** the ipsi- and contralateral muscle as the site of immunisation (SOI) for each vaccine (*n* = 6). Ad alone and BCG alone: contralateral leg received no vaccine, Ad + BCG: each leg received one of the vaccines. Individual cytokine kinetics are shown in Supplementary Fig. 3. **e** Summary of the experimental design for data in **f**). **f** HMDMs were stimulated with Ad and/or BCG for 24 h at the indicated MOI/CFU. IL-1β and TNF secretion in supernatants was measured by ELISA. Representative data from six independent experiments, showing biological replicates (*n* = 3 PBMC donors). Data show the mean + SEM. Two-way ANOVA with Bonferroni’s multiple comparison; *p < 0.05; **p < 0.01; ***p < 0.001; ****p < 0.0001.

Activation of innate immunity by BCG is well documented^20^, and could play a key mechanistic role in the observed effect on the adaptive response to the Ad-vectored vaccine. We profiled the early innate responses to the three vaccination regimens (Ad, BGC, Ad + BCG), by analysing the cytokine secretion patterns at the site of injection (SOI), and systemically, in the serum and the spleen (Fig. 2a). There was a clear difference in systemically induced cytokines between Ad and BCG, with Ad inducing IFN-γ, IFN-α and CXCL-10 secretion, and BCG promoting production of CXCL1 and IL-6 (Fig. 2b, c and Supplementary Fig. 3). This effect was particularly pronounced in the serum. Simultaneous vaccination with Ad and BCG resulted in a broader, merged pattern of secreted cytokines (Fig. 2b and Supplementary Fig. 3), supporting the premise of BCG-promoted adjuvantation through expanding the pro-inflammatory cytokine spectrum. Locally, in the muscle (SOI), additional cytokines were induced by Ad (CCL5) or BCG (CCL2, IL-1β, GM-CSF, and TNF, Fig. 2d and Supplementary Fig. 3), which were not detected systemically. TNF and IL-1β, two important markers of adjuvanticity^22^, were detected only at the SOI but could nevertheless have a downstream influence on the immune response to Ad.

Interestingly, in Ad+BCG vaccinated mice, with Ad and BCG given into the opposite limbs, the vaccinated sites showed lower cytokine secretion compared to the respective single-vaccinated mice (Fig. 2d). This could be the result of one or more processes, such as shared recruitment of circulating immune cells between the two SOIs reducing the cytokine levels at a given site (as previously reported for concurrent infection with multiple pathogens^23^), leukocyte trafficking/rapid lymphatic export of soluble mediators away from the site of injection towards systemic distribution^24^, or inflammation-triggered negative feedback loop locally via, for example, induction of corticosteroids. Nonetheless, likely due to an additive immune response to the two vaccines systemically, this local cytokine reduction did not affect their total circulating levels, either qualitatively or quantitatively, as documented in the serum and the spleen (Fig. 2b, c and Supplementary Fig. 3). Overall, our innate cytokine data are in line with previous findings from immunising with Ad^25^ or BCG^26^ alone, and reveal the induction of a pool of pro-inflammatory cytokines (IFN-γ, IFN-α, IL-6, IL-1β, and TNF), chemo-attractants (CXCL1, CCL2, CCL5, and CXCL-10), and the colony-stimulating factor GM-CSF, when both vaccines are given at the same time.

To corroborate these findings in a human system, we utilised human macrophages derived from peripheral blood CD14^+^ monocytes differentiated using M-CSF. Activation of the NLRP3 inflammasome after in vitro stimulation with Ad, BCG or Ad+BCG (Fig. 2e) was measured as a key indicator of adjuvanticity^22^ and BCG-mediated imprinting^27^. Direct TLR stimulation of myeloid cells induces NFκB signalling, promoting TNF secretion and the expression and post-translational modifications of the NLRP3 inflammasome^22^. TLR priming also strongly induces the expression of pro-IL-1β, ready for inflammasome-mediated processing into the active form. Full inflammasome activation requires a second signal, typically delivered from pathogen or vaccine uptake, leading to the secretion of IL-1β and IL-18, and pro-inflammatory cell death^22^. Interestingly, in vitro combining Ad and BCG at a lower dose of BCG increased amounts of secreted IL-1β, compared to BCG alone (Fig. 2f). A higher dose of BCG apparently saturated the capacity of HMDMs to secrete IL-1β which was not further increased by the addition of Ad (Fig. 2f). Interestingly, using the inflammasome inhibitor MCC950 showed that BCG-induced IL-1β secretion is NLRP3 dependent, while Ad+BCG co-administration engages additional signalling pathways, and is only partially dependent on NLRP3. AdHu5 vector is known as a poor inducer of secreted IL-1β^28^, indicating either a lack of induction of pro-IL-1β, or absence of inflammasome activation. BCG can induce transcriptional upregulation of pro-IL-1β but only limited secretion of the active cytokine, in line with its recognised role as a modest activator of the inflammasome pathway^29^. Our data broadly support this, demonstrating secreted IL-1β in the BCG- but not the Ad-vaccinated muscle (Fig. 2c and Supplementary Fig. 3).

Similarly to IL-1β, TNF secretion, which is induced by TLR signalling and independent of inflammasome activation, was increased in response to Ad+BCG in vitro at a lower BCG dose (Fig. 2f). These findings further demonstrate the additive effect of Ad and BCG in activating the TLRs and the inflammasome in human macrophages. As well-established hallmarks of potent adjuvanticity^22^, engagement and activation of these pathways is likely to be beneficial for the efficacy of both vaccines.

In summary, we demonstrate that combining BCG with a viral-vectored malaria vaccine generates a broader innate immune response and enables protection against malaria challenge. This encouraging finding reflects a context-specific observation, and further studies are needed to determine its applicability to polyclonal, multi-epitope vaccine responses and to the naturally complex malaria antigens in human infection. Simultaneous administration of both vaccines induces a potent inflammatory cytokine profile, capable of supporting protective CD8^+^ T cells in this challenge model. Recent studies in mice have shown that intravenously administered BCG can protect against SARS-CoV-2 via CD4^+^ T cell-mediated IFN-γ secretion, through imprinting of myeloid and epithelial cells for enhanced antiviral immunity^21,30^. In clinical trials, previous administration of BCG has been found to enhance the immune response to RNA- or viral vector-based SARS-CoV-2 vaccines^31,32^. Consequently, our findings may broaden the portfolio of advantageous applications of the BCG vaccine, identifying co-administration of BCG and malaria vaccines as a potentially promising strategy to combat both TB and malaria in double-endemic regions.

## Methods

### Mice and immunisations

For all experiments, mice were purchased from Envigo, UK. Animals were maintained at the Centre for Human Genetics, University of Oxford, housed under specific pathogen-free (SPF) conditions in individually ventilated cages, with constant temperature and humidity and a 12 h light/dark cycle, and in accordance with the recommendations of the UK Animals (Scientific Procedures) Act 1986 and ARRIVE guidelines. Protocols were approved by the University of Oxford Animal Care and Ethical Review Committee for use under Project Licences P9804B4F1 and PP0984913, granted by the UK Home Office.

Anaesthetised (using vaporised IsoFlo^®^) female BALB/c mice (BALB/cOlaHsd), aged 7–10-weeks, were immunised intramuscularly (i.m.) with a total volume of 50 μL in the tibialis muscle. In the initial experiments, BCG was administered intradermally (i.d.) into the ear (2 × 25 μL each site). Comparison with i.m. administration showed no difference in protection against malaria, and BCG was given i.m. in subsequent experiments. Each vaccine dose contained 0.5 × 10^5^ CFU BCG, or 5 × 10^7^ i.u. AdHu5-TIPeGFP. These routes were chosen to mimic the route used to deliver the equivalent vaccines in people, e.g., BCG is routinely given i.d. in the clinic. At the end of the experiments, mice were euthanised using CO_2_ followed by cervical dislocation.

### Sporozoite production

*Plasmodium berghei* (ANKA strain clone 234) sporozoites were produced as previously described^33^. Starved female *Anopheles stephensi* mosquitoes were fed for approximately 10 min on BALB/c mice infected with *P. berghei* parasites. For the mosquito feeding, mice were anaesthetised by i.m. injection of 50 μL Ketamine/Xylazine mix (40 mg/ml Ketamine, 0.4% w/v Xylazine).

The mosquitoes were then maintained on Fructose/PABA solution at 19–21 °C in a humidified incubator on a 12 h light/dark cycle. Approximately 21 days after feeding, the mosquitoes were dissected, and the salivary glands were removed. While keeping on ice, the glands were placed in RPMI-1640 and disrupted with a tissue homogeniser to release the sporozoites. The sporozoites were counted using a haemocytometer, while keeping them on ice to preserve viability, and immediately proceeded to malaria challenge.

### Malaria challenge

Malaria challenge was performed as described previously^33^. For all experiments, 1000 *P. berghei* sporozoites (described above) were injected intravenously without anaesthesia in a total volume of 100 μL into the lateral tail vein of each mouse. From day 5 post challenge, the mice were monitored for infection by thin-film blood smear (fixed in methanol and stained in 10% Giemsa for 30 min). Mice were sacrificed when >1% parasitaemia was observed. If no parasites were detected on day 12 after challenge, mice were considered sterilely protected.

### Ex vivo IFN-γ splenocyte ELISpot

ELISpot assays were performed using the IFN-γ ELISpot (kit 3321-2A, Mabtech, UK) as described previously^33^. In brief, two weeks after immunisation, mice were culled and spleens removed. Single cell suspensions were prepared by homogenising and straining the spleens, followed by brief incubation in ACK lysis buffer to remove erythrocytes. Cells were washed with PBS, pelleted, resuspended in complete α-MEM and counted using the CASY cell counter. MultiScreen-IP 0.45 μm sterile plates were coated with 5 μg/ml rat anti-mouse interferon gamma mAb AN18 diluted in carbonate-bicarbonate buffer overnight at 4 °C. Plates were then blocked for at least 1 h at 37 °C with complete α-MEM. Cells were plated and stimulated for 18–20 h with 1 μg/mL of Pb9 peptide. Plates were washed and incubated with 1 μg/mL biotinylated anti-mouse-IFNγ mAb R46A2, followed by incubation with 1 μg/mL streptavidin alkaline phosphatase. Spots were developed by the addition of 50 μL per well of colour development buffer and counted using ELISpot software (AID). Results are expressed as spot-forming cells (SFC) per million splenocytes after subtracting background responses in unstimulated wells.

### Flow cytometry

Phenotypic analysis of CD8^+^ T cells was performed by surface marker staining using the following fluorescently labelled antibodies: PE-Cy7-CD11a (BD Bioscience cat #558191), PE-CD186 (BioLegend cat #151104), BV711-CD69 (Biolegend cat #104537), BV650-CD44 (BioLegend cat #103049), BV605-CD62L (BioLegend cat #104437), BV421-KLRG1 (BioLegend cat #138413), AF700-CD8 (BioLegend cat #100729). Non-specific Ab binding was prevented by incubation with anti-CD16/CD32 Fcγ III/II Receptor (BD Pharmingen, UK) prior to the surface staining. The Pb9 tetramer was provided by the NIH tetramer facility (MHC tetramer core facility, Emory University Vaccine Center, Atlanta, USA) using the SYIPSAEKI peptide. Live and dead cells were distinguished using LIVE/DEAD^TM^ Fixable Aqua Dead Stain Kit (Thermo Fisher Scientific, UK). To define subpopulation distribution within the Pb9^+^ compartment (Fig. 1d, e) cells were gated on singlets, size, CD8^+^, Pb9^+^, and subsequent definition of subpopulations as depicted in Fig. Supplementary Fig. 1d without gating on CD44^+^/CD11a^+^ as Pb9^+^ cells were generally positive for these markers. Flow cytometric analyses were performed on LSRII (BD Biosciences, UK) and data were analysed with FlowJo software.

For B cell profiling, freshly harvested splenocytes were seeded in 96-well plates at a density of ~1.5 × 10^6^ cells per well in chilled FACS buffer. Cells were washed and stained with diluted nIR Zombie L/D stain (Biolegend cat #423105) and incubated for 15 min at room temperature. After incubation, cells were washed and stained with a cocktail of the following antibodies diluted in FACS buffer: BV421-CD19 (Biolegend cat #115549), BV605-B220 (Biolegend cat #130244), BV786-CD95 (BD Bioscience cat #740716), PerCPCy5.5-GL7 (BioLegend cat #144609), PECy5-CD38 (Invitrogen cat #15-0381-82), PE-Cy7-CD138 (BioLegend cat #142513), AF700-IgD (BioLegend cat #405729), APCCy7-CD3 (BioLegend cat #100222), APCCy7-F4/80 (BioLegend cat #123117). Cells were subsequently washed and transferred to FACS tubes for flow cytometric analyses on a Fortessa (BD Biosciences, UK).

### Total IgG ELISA

ELISAs to detect antibodies against eGFP (expressed by AdHu5-TIPeGFP) or total BCG were performed as previously described^33^. Serum was obtained by collecting blood from the lateral tail vein in a microcuvette tube. Blood was allowed to clot at 4 °C overnight before centrifugation at 13,000 rpm for 4 min, and sera were removed and stored at −20 °C until use. For total IgG ELISAs, Nunc-Immuno Maxisorp 96-well plates were coated with 1 μg/mL eGFP in carbonate-bicarbonate coating buffer or 10^5^ CFU BCG per well overnight at 4 °C. Plates were washed with PBS-Tween (0.05% v/v) and blocked with 2% BSA in PBS-Tween for 1 h at RT. Sera were diluted appropriately between 1:200 and 1:3200 (depending on Ag and time point) in 1% BSA in PBS-Tween and added to the plate in duplicates. Plates were incubated for 2 h at room temperature and then washed as before. Fc-specific goat anti-mouse IgG conjugated to alkaline phosphatase (AP) (1 in 5000, Sigma-Aldrich, A1418) was added for 1 h at room temperature (eGFP) or overnight at 4 °C (BCG). Following a final wash, plates were developed by adding *p*-nitrophenylphosphate at 1 mg/mL in diethanolamine buffer, and OD was read at 405 nm. Total IgG concentrations against eGFP or BCG in sera were calculated by interpolation against a standard curve generated from a serum pool of previously AdHu5-TIPeGFP or BCG immunised mice. Sera from naive mice were used as a negative control.

### IgG subclass ELISA

To assess the quality of the antibody response against eGFP (expressed by AdHu5-TIPeGFP), IgG subclass ELISAs were performed as previously described^34^. MaxiSorp plates (Nunc) were coated with 1 μg/mL eGFP in carbonate-bicarbonate coating buffer overnight at 4 °C prior to washing in PBS-Tween (0.05% v/v) and blocking with 2% BSA in PBS-Tween for 1 h at RT. For the detection of IgG subclasses, all serum samples were diluted to 1 ELISA-unit of total anti-eGFP IgG in 1% BSA in PBS-Tween and added in duplicates. Plates were incubated for 2 h at 37 °C and then washed as before. Afterwards, AP-conjugated anti-mouse IgG subclass-specific secondary antibodies IgG1 (1 in 4000, Southern Biotech), IgG2a (1 in 4000, Southern Biotech), IgG2b (1 in 4000, Southern Biotech) or IgG3 (1 in 1000, Abcam) were added and incubated for 1 h at 37 °C. Following a final wash, plates were developed by adding *p*-nitrophenylphosphate at 1 mg/mL in diethanolamine buffer, and OD was read at 405 nm. The results of the IgG subclass ELISAs are presented using optical density values.

### T_H_1/T_H_2 index

To evaluate the T_H_1/T_H_2 index, serum titres of IgG1, IgG2a, IgG2b, and IgG3 were determined as described above. Afterward, the index was calculated as ([IgG2a or IgG2c + IgG3]/2)/(IgG1) as previously described^35^. Increased T_H_1/T_H_2 index indicates T_H_1 skewed immune responses.

## ELISA for detection of cytokines in serum, spleen and muscle (SOI)

Tissue cytokines were assessed as described previously^36^. To detect innate cytokines in serum, spleen and muscle (SOI) samples, mice were immunised with AdHu5-TIPeGFP and/or BCG as described above. At specific time points, mice were sacrificed, and tissues were harvested. Blood samples were allowed to clot for 2 h before centrifugation at 13,000 rpm for 4 min; sera were stored at −80 °C until use. Muscle and spleen samples were weighed, transferred into PBS supplemented with protease inhibitor (Roche, 11873580001), and dissociated in gentleMACS M Tubes using the gentleMACS Octo Dissociator. Cell suspensions were stored at −80 °C. Before use, samples were thawed, clarified by centrifugation at 10,000 rpm for 5 min, and supernatants analysed for cytokines and chemokines using the mouse MU Anti-Virus Response Panel flow assay kit (LEGENDplex, BioLegend) according to the manufacturer’s instructions. The LSR Fortessa (BD) flow cytometer was used to assess the fluorescence intensity of beads, and data were analysed using LEGENDplex Data Analysis Software Suite.

## Human monocyte-derived macrophages (HMDM)

Assays on human monocyte-derived macrophages were performed as previously described^36^. In brief, peripheral blood mononuclear cells were isolated using Ficoll gradient from healthy donors from the NHS Oxford blood bank (REC approval 11/H0711/7). Magnetic beads (eBioscience, 8802-6834-74) were used to positively select CD14^+^ monocytes, which were differentiated into macrophages by culturing for 7 days with M-CSF (100 ng/mL, BioLegend, 574808). Cells were cultured in RPMI (Fisher, 21870076) with 10% FBS (Life Technologies, 16000044), 25 mM HEPES buffer (Fisher, 15630056) and 1x Pen/Strep/Glutamine (Fisher, 10378016) at 37 °C with 5% CO_2_, and supplemented with fresh media containing 100 ng/mL M-CSF on day 2 and day 5. After day 7 of differentiation, cells were re-plated (70,000 cells per well in flat-bottom 96-well plates in 100 μL complete RPMI), and on day 8, cells were stimulated for inflammasome-activation experiments. Differentiated HMDMs were plated at a density of 0.7 × 10^6^ cells/mL in complete RPMI. Before stimulation, complete RPMI is replaced with antibiotic-free RPMI (supplemented with 10% FBS, 25 mM HEPES buffer, and 1x GlutaMAX (Fisher, 35-050-061)). MCC950 (Sigma-Aldrich, 5381200001) was added at 10 µM 30 min before stimulation. Cells are then stimulated for 24 h with AdHu5-TIPeGFP and/or BCG at concentrations indicated in the Figures. After cell stimulation, cell supernatants were collected for analysis. Secretion of IL-1β and TNF was measured in cell-free supernatants using ELISA (Thermo Fisher, 88-7261-77 and 88-7346-88) according to the manufacturer’s instructions.

### Statistical analysis

Statistical analysis was performed using Prism software (V10). Statistical tests applied, and pairwise comparisons with correction for multiple testing are indicated in the Figure legends. In all experiments ^∗^*p* < 0.05, ^∗∗^*p* < 0.01, ^∗∗∗^*p* < 0.001, and ^∗∗∗∗^*p* < 0.0001. Non-significant differences (n.s.) are generally not indicated.

## Supplementary information

Supplementary Information

## Data Availability

All data generated or analysed during this study are included in this published article (and its supplementary information ﬁles).
