# Supplementary material for: BCG enables protection against malaria through adjuvanting an adenovirus-vectored vaccine
Source: NPJ Vaccines. 2026 Jun 24;11:191. doi: 10.1038/s41541-026-01506-9 (PMC13654936; doi:10.1038/s41541-026-01506-9)
Supplement: Supplementary file 1 [file 41541_2026_1506_MOESM1_ESM.pdf]

Supplementary Material

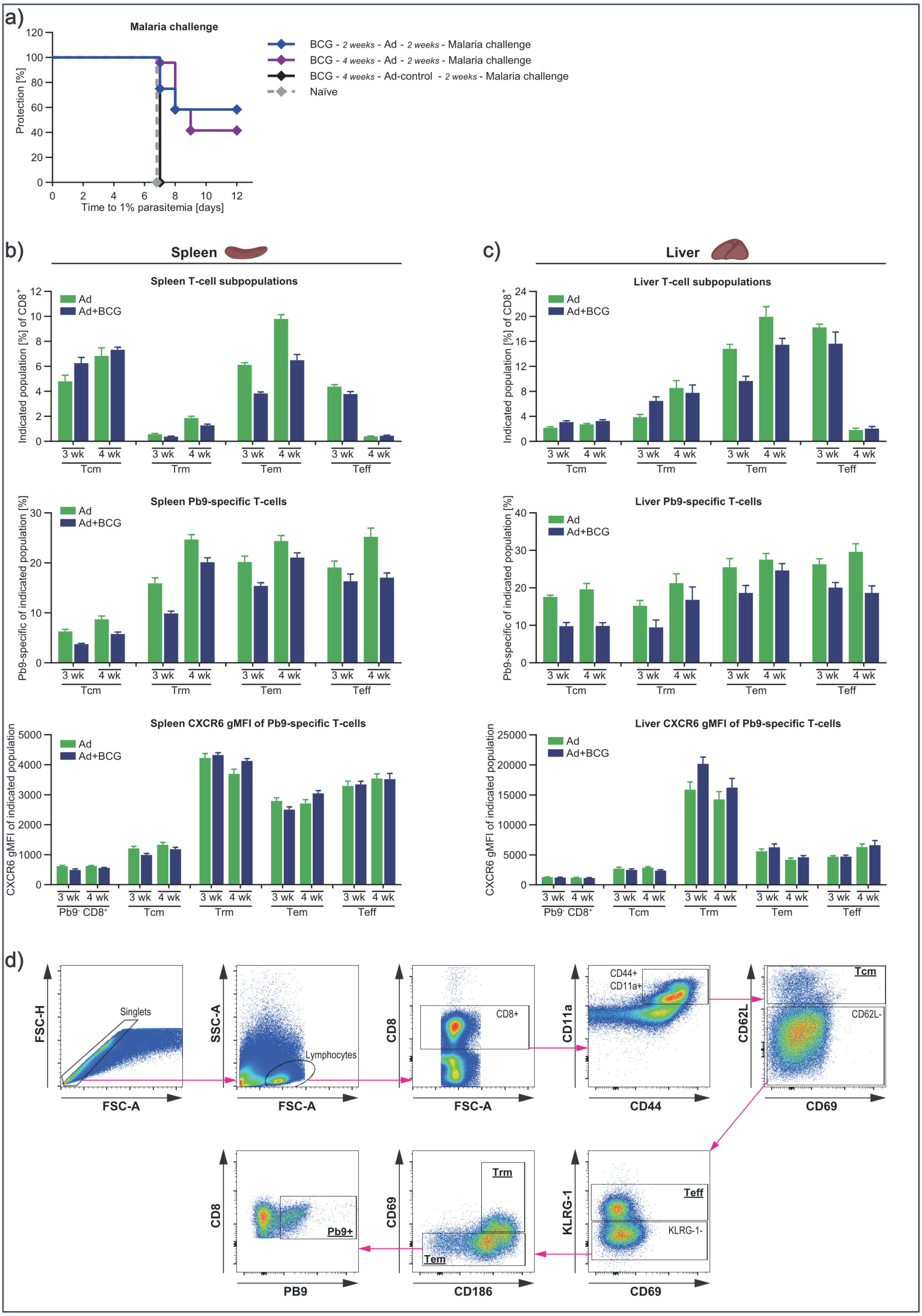

Supplementary Figure 1. Staggered immunisation with BCG and Ad enables partial protection against malaria in mice.

(a) *Survival post-malaria challenge: BALB/c mice were vaccinated with a BCG prime Ad boost regimen at indicated intervals and challenged with malaria 2 weeks post Ad immunisation. Parasitaemia was assessed by daily blood smears, with 1% parasitaemia taken as the end-point (n ≥ 8).*

(b and c) *T cell phenotyping of splenic and hepatic cells by FACS after vaccination and harvest as described in 1a) (n = 6). Top panels show the distribution of T cell subpopulations within the $CD8^+$ T cell compartment. Middle panels show relative numbers of Pb9-specific cells within T cell subpopulations. Bottom panels show the gMFI of the liver homing marker CXCR6 for Pb9-specific T cells and $Pb9^-$ $CD8^+$ T cells.*

(d) *Gating strategy for T cell FACS data shown in this Figure and Figure 1.*

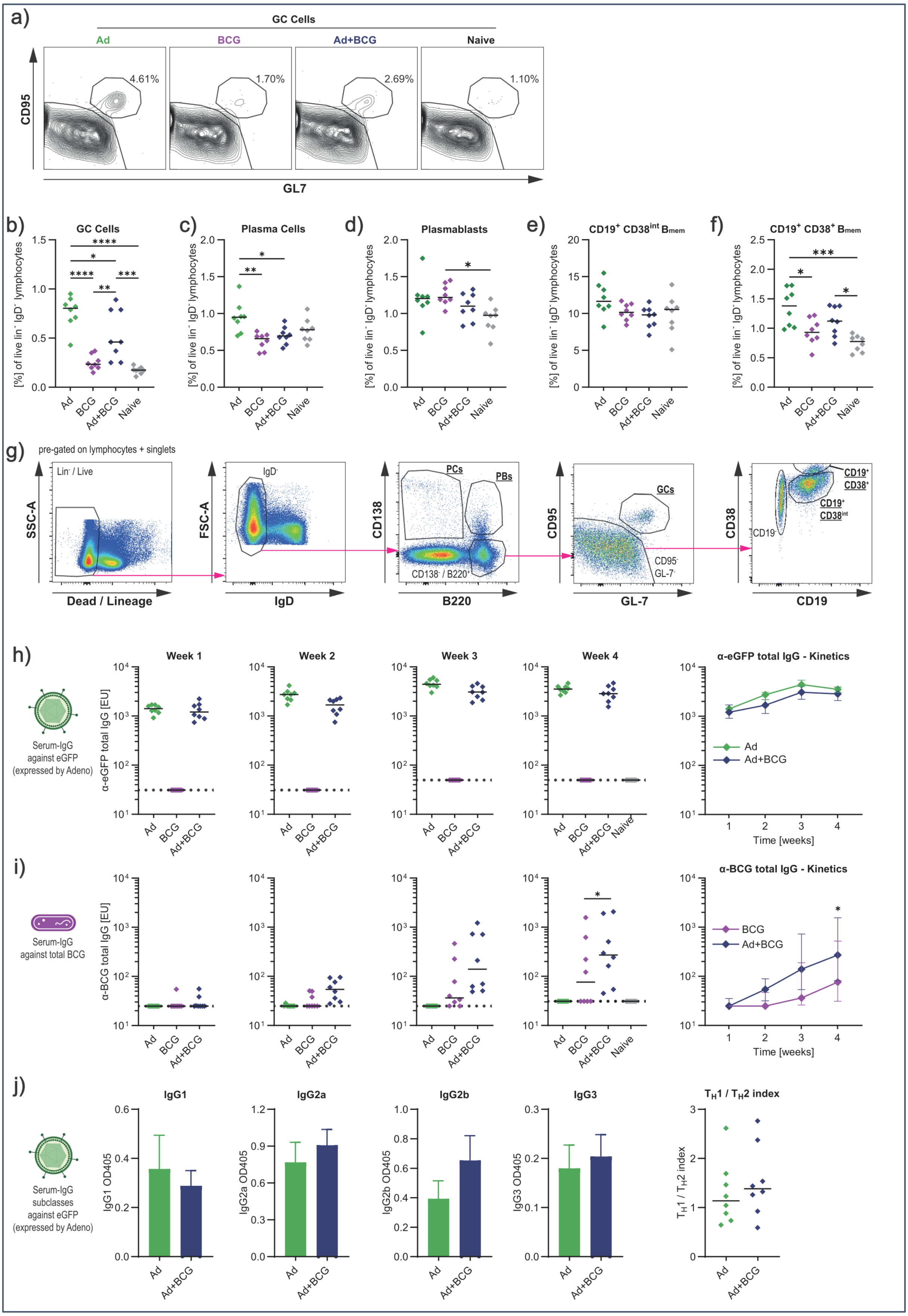

Supplementary Figure 2. Ad and BCG co-administration modulates the B cell compartment and antibody isotype profiles.

***(a-f)** Total B cell phenotyping of splenocytes after vaccination and harvest (as in Figure 1a).*

***(a)** Representative FACS plots of $CD95^+$ $GL7^+$ germinal centre B cell populations, showing percentages of live IgD^-, lin^-, $CD138^-$, $B220^+$ lymphocytes.*

(b) *Quantification of data presented in (a). Percentages of live* IgD^-*, lin*$^-$ *lymphocytes. Each symbol represents an individual mouse (n = 8).*

(b-f) *Quantification of indicated B cells populations after vaccination (n = 8). Median + replicates; one-way ANOVA with Bonferroni's correction for multiple comparisons; *p < 0.05; **p < 0.01; ***p < 0.001; ****p < 0.0001.*

(g) *Gating strategy for B cell FACS data.*

(h) *Serum anti-GFP IgG titres were measured by ELISA at the indicated time-points post immunisation; GFP is encoded by the Ad construct and used as a surrogate vaccine antigen. Each symbol represents an individual mouse (n = 8). Dashed line indicates the limit of detection. The panel on the right shows the anti-GFP antibody (Ab) titres over 4 weeks following vaccination.*

(i) *Serum anti-BCG IgG titres were measured by ELISA at the indicated time-points post immunisation. Each symbol represents an individual mouse (n = 8). Dashed line indicates the limit of detection. The panel on the right shows the anti-BCG Ab titres over 4 weeks post-vaccination. Testing for seroconversion at week 4 using Barnard's Unconditional Test; *p < 0.05.*

(j) *Serum anti-GFP IgG subclasses by proportional ELISA (Mean + SEM).* T_H1/T_H2 *index of IgG subclasses calculated as ([IgG2a + IgG3]/2)/IgG1. Increased* T_H1/T_H2 *index indicates* T_H1*-skewed immune response (median + replicates).*

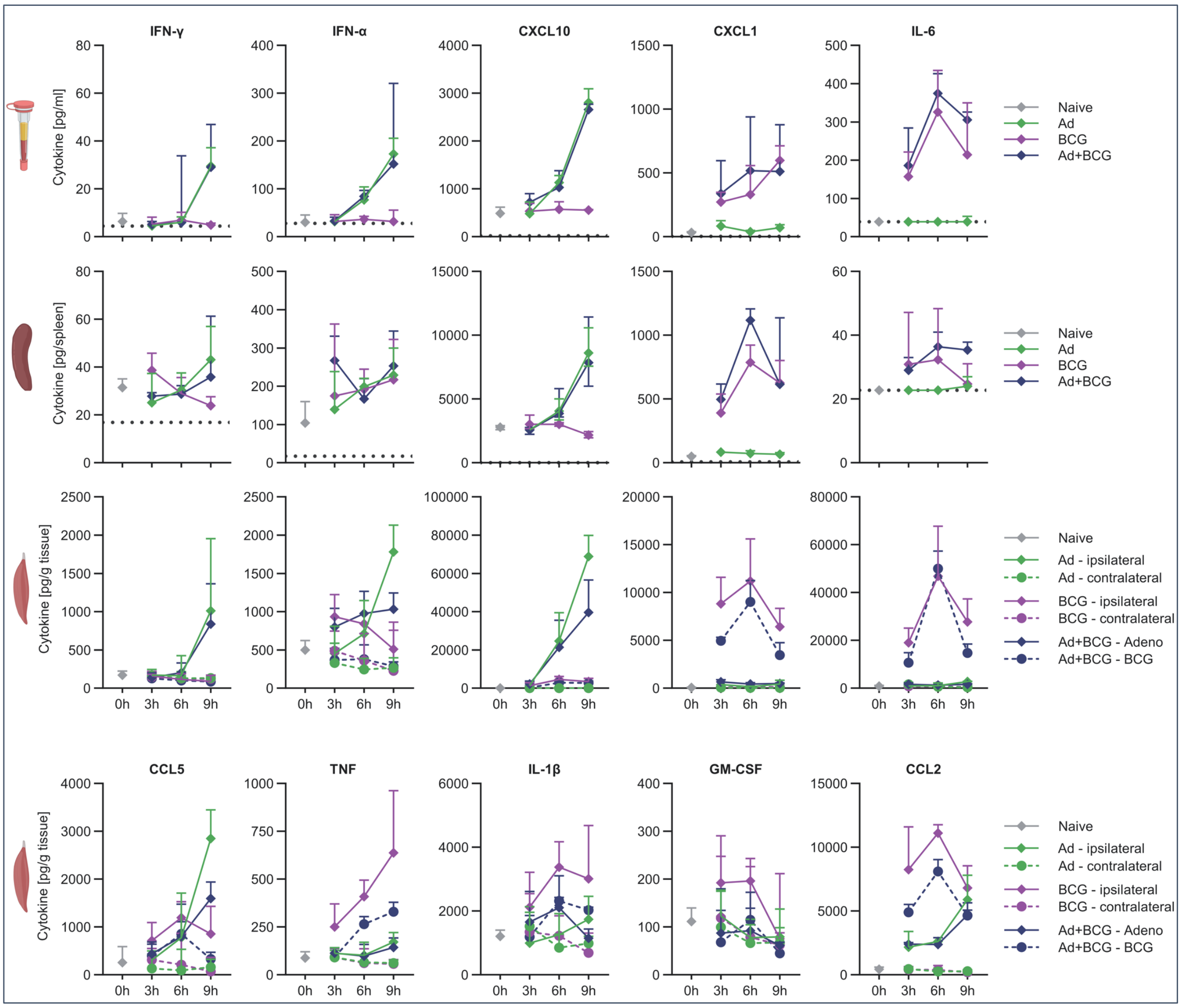

Supplementary Figure 3. Innate cytokine kinetics post Ad and BCG immunisation

Detailed representation of data shown in Figure 2b – d. Vaccine dose per mouse: 0.5x10^5 CFU for BCG and 5x10^7 i.u. for AdHu5-TIPeGFP) (n = 6). In the Ad+BCG group, Ad was injected into the right leg and BCG into the left leg. Indicated cytokines were measured using LEGENDplex^{TM} MU Anti-Virus Response Panel. Graphs show the cytokine levels detected in the serum, spleen, and the site of immunisation (SOI) in the ipsi- and contra-lateral muscles for each vaccine (Ad alone and BCG alone: contralateral leg received no vaccine, Ad+BCG: each leg received one of the vaccines).
